# Controversias en neuroinmunología: esclerosis múltiple, vacunación, SARS-CoV-2 y otros dilemas

**DOI:** 10.7705/biomedica.6366

**Published:** 2022-10-31

**Authors:** Saúl Reyes-Niño, Jaime Eduardo Rodríguez-Orozco, Habib Georges Moutran-Barroso, Hellen Kreinter-Rosembaun, Mariana Gaviria-Carrillo, Vanessa Salej-Durán, Julián Mancera-Charry, Ana Claudia Villegas, David Cuéllar-Giraldo, Juan Sebastián Torres-Sandoval, Ángela Gómez-Mazuera, Arístides Duque-Samper, Jaime Toro-Gómez

**Affiliations:** 1 Departamento de Neurología, Fundación Santa Fe de Bogotá, Bogotá, D.C., Colombia Departamento de Neurología Fundación Santa Fe de Bogotá Bogotá D.C Colombia; 2 Programa de Residente en Neurología Clínica, Universidad El Bosque, Bogotá, D.C., Colombia Universidad El Bosque Universidad El Bosque Bogotá D.C Colombia; 3 Grupo NeURos, Escuela de Medicina y Ciencias de la Salud, Universidad del Rosario, Bogotá, D.C., Colombia Universidad del Rosario Grupo NeURos Escuela de Medicina y Ciencias de la Salud Universidad del Rosario Bogotá D.C Colombia

**Keywords:** esclerosis múltiple, síndrome de Guillain-Barré, coronavirus, polirradiculoneuropatía crónica inflamatoria desmielinizante, sarcoidosis, vacunas, natalizumab, Multiple sclerosis, Guillain-Barré syndrome, coronavirus, polyradiculoneuropathy, chronic inflammatory demyelinating, sarcoidosis, vaccines, natalizumab

## Abstract

La neuroinmunología es una disciplina que cada vez amplía más sus horizontes en la comprensión de las enfermedades neurológicas. Contemporáneamente, y a la luz de los nexos fisiopatológicos de las enfermedades neurológicas y la inmunología, se han planteado enfoques diagnósticos y terapéuticos específicos. A pesar de los importantes avances de esta disciplina, existen múltiples dilemas que le conciernen y se filtran en la práctica clínica.

En esta revisión, se presentan y discuten 15 controversias, las cuales se construyen con la información clínica disponible más actualizada. Los temas incluidos son: disminución de esteroides en recaídas de esclerosis múltiple; recomendaciones terapéuticas en esclerosis múltiple a la luz de la pandemia por el SARS-CoV-2; evidencia de vacunación en esclerosis múltiple y en otras enfermedades desmielinizantes; panorama actual del síndrome clínico y radiológico aislado; y fallas terapéuticas en esclerosis múltiple; además, criterios para suspender las terapias modificadoras de la enfermedad; evidencia del manejo en recaídas leves; recomendaciones para la profilaxis contra *Strongyloides stercolaris;* utilidad de un segundo ciclo de inmunoglobulina en el síndrome de Guillain-Barré; criterios para diferenciar una polineuropatía crónica desmielinizante inflamatoria de inicio agudo de un síndrome de Guillain-Barré y, utilidad de la enzima convertidora de angiotensina en neurosarcoidosis. En cada una de las controversias, se presenta la problemática general y se ofrecen recomendaciones específicas que pueden adoptarse en la práctica clínica diaria.

## Abstract (en)

Neuroimmunology is a discipline that increasingly broadens its horizons in the understanding of neurological diseases. At the same time, and in front of the pathophysiological links of neurological diseases and immunology, specific diagnostic and therapeutic approaches have been proposed. Despite the important advances in this discipline, there are multiple dilemmas that concern and filter into clinical practice. This article presents 15 controversies and a discussion about them, which are built with the most up-to-date evidence available. The topics included in this review are: steroid decline in relapses of multiple sclerosis; therapeutic recommendations in MS in light of the SARS-CoV-2 pandemic; evidence of vaccination in multiple sclerosis and other demyelinating diseases; overview current situation of isolated clinical and radiological syndrome; therapeutic failure in multiple sclerosis, as well as criteria for suspension of disease-modifying therapies; evidence of the management of mild relapses in multiple sclerosis; recommendations for prophylaxis against *Strongyloides stercolaris;* usefulness of a second course of immunoglobulin in the Guillain-Barré syndrome; criteria to differentiate an acute-onset inflammatory demyelinating chronic polyneuropathy versus Guillain-Barré syndrome; and, the utility of angiotensin-converting enzyme in neurosarcoidosis. In each of the controversies, the general problem is presented, and specific recommendations are offered that can be adopted in daily clinical practice.

## Controversias en Neuroinmunología

En los pacientes con esclerosis múltiple que presentan recaídas y han recibido pulsos intravenosos de metilprednisolona, ¿se deben disminuir gradualmente los esteroides?

En el estudio ONTT se demostró un beneficio en la disminución de los esteroides en un primer episodio de neuritis óptica.

En este estudio, aleatorizado, controlado y doble ciego, se incluyeron pacientes entre los 18 y los 46 años con un primer episodio de neuritis óptica, y se distribuyeron en tres grupos con el fin de evaluar los resultados en la visión: el grupo 1, con 250 mg de metilprednisolona intravenosa cada 6 horas por 3 días más 1 mg/kg/día de prednisolona oral por 11 días; el grupo 2, con 1 mg/kg/día de prednisolona oral por 14 días, y el grupo 3, con placebo.

Los sujetos del grupo 1 presentaron una recuperación más rápida en los campos visuales y la visión de contraste, en comparación con los grupos 2 y 3. Teniendo en cuenta lo anterior, aquellos pacientes que presentan neuritis óptica como primer síndrome clínico de la esclerosis múltiple, deberían recibir metilprednisolona intravenosa seguida de prednisolona oral, con el fin de acelerar la recuperación de la visión ^1^.

En contraste, para los pacientes que ya tienen diagnóstico de esclerosis múltiple y sufren una recaída clínica, los datos disponibles hasta el momento han demostrado que la disminución del esteroide oral después de la metilprednisolona intravenosa no mejora la discapacidad ni acelera la recuperación. Por esta razón, en estos casos, los esteroides orales no producen ningún beneficio adicional después de los pulsos de metilprednisolona ^2^.

Además, la metilprednisolona intravenosa durante las recaídas de la esclerosis múltiple no genera una supresión permanente del eje hipotálamo-hipófiso-suprarrenal. Levic, *et al.,* lograron demostrar que los pacientes con una dosis de 1 gramo diario durante siete días presentan una supresión transitoria del eje, el cual logra recuperarse de forma espontánea después de los pulsos sin necesidad de tratamientos adicionales. Por esta razón, no son necesarios los esteroides orales suplementarios ^3^.

## ¿Cuáles son las terapias modificadoras de la enfermedad que se asocian con mayor gravedad de la COVID-19? ¿Se deberían evitar o suspender?

Con el inicio de la pandemia por COVID-19, comenzaron a surgir varios interrogantes en relación con las terapias modificadoras de la enfermedad para la esclerosis múltiple.

En primer lugar, despertaba preocupación el hecho de que algunas de ellas (por ejemplo, alemtuzumab y ocrelizumab) producen linfopenia como un efecto particular. Además, los tratamientos que se administran por vía intravenosa implican el desplazamiento frecuente de los pacientes a los centros de salud, lo cual aumenta potencialmente su exposición y el riesgo de contagio. De forma similar, algunos de los tratamientos requieren un control frecuente con pruebas séricas que también implican asistir con frecuencia a los centros de salud.

En respuesta a la creciente incertidumbre, se establecieron varios registros poblacionales a nivel global, de los cuales surgen los datos con los que contamos hasta la fecha.

En abril de 2020, Sormani, *et al.,* publicaron su experiencia con el registro poblacional italiano MuSC-19. Este incluyó 784 pacientes con esclerosis múltiple y fuerte sospecha diagnóstica o diagnóstico definitivo de COVID-19. El resultado primario evaluado fue la gravedad de la COVID-19, demostrada por uno o más de los siguientes factores: muerte, necesidad de traslado a la unidad de cuidados intensivos, neumonía u hospitalización.

Entre los hallazgos más significativos, las terapias anti-CD20 (ocrelizumab y rituximab) se asociaron con un mayor riesgo de gravedad de la COVID-19 (OR=2,59; IC_95%_ 1,43-4,67; p<0,001). Además, se identificaron como factores de riesgo para enfermedad grave: la edad (OR=1,06; IC_95%_ 1,03-1,08; p<0,001), la forma progresiva de la enfermedad (OR=2,07°; IC_95%_ 1,22-3,50; p<0,001) y el uso reciente (en el último mes) de metilprednisolona (OR=6,03; IC_95%_ 2,2-6,54; p<0,001) ^4^.

Posteriormente, Louapre, *et al.,* publicaron un estudio retrospectivo de cohorte realizado a partir del registro francés COVISEP, en el cual se incluyeron 347 pacientes con esclerosis múltiple y fuerte sospecha diagnóstica o diagnóstico definitivo de COVID-19.

Para evaluar el resultado primario de gravedad de la COVID-19, diseñaron una escala ordinal de 1 a 7, en la cual 1 correspondía a "no hospitalizado, no limitación funcional" y 7, a "muerte" Establecieron un punto de corte de 3 que significaba "hospitalizado sin necesidad de oxígeno suplementario.

Los resultados no mostraron asociación de ninguna terapia en particular con una mayor gravedad de la COVID-19. De hecho, los pacientes que no recibían terapias modificadoras de la enfermedad tuvieron peores resultados (incluyendo mortalidad), en comparación con aquellos que sí recibían tratamiento (46,0 *Vs.* 15,5 %; p<0,001). Además, se encontró que la edad (OR=1,9; IC_95%_ 1,4-2,5), la escala EDSS (OR=6,3; IC95% 2,8-14,4) y la obesidad (OR=3,0; IC_95%_1,0-8,7), eran factores de riesgo independientes para un puntaje de gravedad de la COVID-19 de 3 o más ^5^.

Durante la octava asamblea virtual de la ACTRIMS-ECTRIMS, se publicaron los datos preliminares obtenidos a partir del *MS Global Data Sharing Initiative:* un canal de recolección y unificación de datos, en el cual participan actualmente hasta 23 registros poblacionales a nivel global. Se tomaron datos de 1.540 pacientes con esclerosis múltiple, con sospecha diagnóstica o con diagnóstico confirmado de COVID-19.

En este estudio se confirmaron algunos hallazgos de los estudios previos: la edad avanzada, ser hombre, la discapacidad y la forma progresiva de la enfermedad, se asociaron con peores consecuencias de la COVID-19. Además, se concluyó que las terapias modificadoras de la enfermedad anti-CD20, particularmente rituximab y en menor medida ocrelizumab, se asociaron con peores consecuencias para COVID-19.

El rituximab se asoció de forma estadísticamente significativa con mayor frecuencia de hospitalizaciones, ingreso en la unidad de cuidados intensivos y mayor necesidad de asistencia respiratoria mecánica. El ocrelizumab no alcanzó significancia estadística para admisión hospitalaria o asistencia respiratoria, pero sí para admisión en la unidad de cuidados intensivos. Ninguno de los dos medicamentos anti-CD20 se asoció con muerte o comorbilidades ^6^.

Recientemente, en un panel internacional de expertos, se publicó una serie de recomendaciones sobre las terapias modificadoras de la enfermedad durante la pandemia y después de ella ^7^.

Estas recomendaciones se basaron en la mejor información disponible hasta el momento y se adoptan para los fines de este manuscrito:

- cualquier decisión de iniciar o administrar una terapia modificadora de la enfermedad debe evaluarse a la luz del estado epidemiológico local de la pandemia y las características individuales del paciente;
- los interferones, el acetato de glatiramer y la teriflunomida, se pueden prescribir de forma usual durante la pandemia;
- se recomienda la administración de natalizumab en esquema de dosis extendida (cada 5 a 6 semanas), para disminuir el número de viajes a los centros de infusión y, potencialmente, mitigar el riesgo de exposición al SARS-CoV-2;
- el dimetil fumarato, el fingolimod y la cladribina también pueden prescribirse de forma usual durante la pandemia, pero, si el paciente está gravemente linfopénico por efecto de la medicación, se recomienda ser particularmente cuidadoso con las medidas de protección para evitar el contagio;
- las terapias anti-CD20 pueden ofrecerse a pacientes con esclerosis múltiple muy activa, siguiendo estrictas medidas de prevención contra el contagio; sin embargo, puede considerarse otra de estas terapias modificadoras muy efectiva con un perfil más favorable en términos de consecuencias de la COVID-19;
- el alemtuzumab puede ofrecerse a pacientes con esclerosis múltiple muy activa y se deben considerar estrictas medidas de prevención contra el contagio en aquellos sujetos con linfopenia. Sin embargo, puede considerarse otra terapia modificadora muy efectiva con un perfil mejor establecido en términos de consecuencias de la COVID-19;
- no se recomiendan terapias de reconstitución inmunológica como la mitoxantrona y el trasplante de células madre hematopoyéticas cuando el riesgo de COVID-19 es grande.

## ¿Las terapias modificadoras de la enfermedad alteran la eficacia de las vacunas?

Algunas de estas terapias pueden influir en la respuesta inmunológica a las inmunizaciones ^8^^,^^9^. Hasta la fecha, no se han notificado problemas de eficacia para el interferón, el dimetil fumarato o la teriflunomida, en relación con las vacunas en general ^8^^,^^10^^-^^12^.

Para el acetato de glatiramer y el natalizumab, la información disponible es menos concluyente; algunos estudios demuestran que la vacuna inactivada contra la influenza puede ser menos efectiva en pacientes con esclerosis múltiple que utilizan estas terapias modificadoras de la enfermedad ^8^. Se ha descrito una respuesta inmunológica reducida a algunas vacunas en pacientes tratados con moduladores del esfingosina-1-fosfato, como lo son el fingolimod y el siponimod ^13^^,^^14^. También, se ha documentado una respuesta humoral reducida a las vacunas, pero no ausente, en pacientes tratados con ocrelizumab ^15^.

Finalmente, aquellos en tratamiento de reconstitución inmunológica (alemtuzumab, cladribina o trasplante de células madre hematopoyéticas) que han reconstituido su sistema inmunológico, deberían poder reaccionar a las vacunas, teniendo en cuenta las siguientes consideraciones:

La vacunación dentro de los primeros seis meses de tratamiento con alemtuzumab puede resultar en una menor proporción de casos que responden ^16^.

Datos de los estudios MAGNIFY-MS y CLOCK-MS sugieren que los pacientes que reciben cladribina pueden generar respuestas a las vacunas contra la influenza y la varicela-zóster, independientemente del recuento de linfocitos ^17^.

Una proporción sustancial de pacientes que han recibido trasplante de células madre hematopoyéticas, pueden responder a las vacunas desde los tres meses de instaurado el tratamiento ^18^.

## ¿Qué tan temprano se debe empezar a considerar una falla terapéutica en pacientes con esclerosis múltiple que han iniciado recientemente una terapia modificadora de la enfermedad?

Uno de los principales objetivos terapéuticos en la esclerosis múltiple es controlar la actividad de la enfermedad y retrasar su progresión.

La definición de falla terapéutica en esclerosis múltiple, en particular aquella utilizada en las pruebas clínicas, ha sido criticada por los cortos tiempos en que se evalúa la reacción a la terapia modificadora de la enfermedad. En general, el tiempo mínimo para empezar a pensar en falla terapéutica es de seis meses después de iniciar dicha terapia ^19^.

Sin embargo, esta definición carece de especificidad y no refleja el tiempo de inicio de acción de todos los fármacos usados en esta enfermedad. La adopción de un periodo estándar de seis meses para evaluar la respuesta a la terapia modificadora de la enfermedad, podría afectar la toma de decisiones e impactar sobre las consecuencias clínicas de los pacientes con esclerosis múltiple a largo plazo ^20^.

Se hace imperativo, entonces, estar familiarizado con los tiempos adecuados para solicitar estudios de imágenes diagnósticas de control en aquellos pacientes que recientemente han iniciado una terapia modificadora de la enfermedad.

El primer examen de imágenes después de iniciarla, se practica usualmente dentro de los primeros 3 a 6 meses, teniendo en cuenta las siguientes consideraciones:

- La aparición de nuevas lesiones en T2 que no se realzan con el medio de contraste en comparación con la resonancia magnética (RM) previa al inicio del medicamento, no necesariamente refleja falla del tratamiento, pues estas lesiones pudieron haber aparecido en cualquier momento entre la toma de la RM previa y el inicio del efecto del medicamento.
- La gran mayoría de las lesiones nuevas se realzan con medio de contraste durante un periodo menor de dos meses y su interpretación se debe hacer con cautela, analizando el momento de la práctica de la RM frente a los tiempos de acción de las terapias modificadoras de la enfermedad ^21^. En general, se recomienda que esta primera imagen realizada poco después de iniciar la terapia modificadora se utilice como punto de referencia para comparar las RM subsecuentes ^19^.

A partir de los puntos anteriormente mencionados, surge la siguiente pregunta: ¿Cuándo se inicia el efecto de las terapias modificadoras de la enfermedad? En un estudio reciente, usaron la información de dos registros de pacientes con esclerosis múltiple que sumaron 125.000 participantes; trazaron una curva de densidades sobre la incidencia de recaídas o la detección de progresión de discapacidad, antes de iniciado el tratamiento y después de haberlo hecho.

De esta forma, determinaron cuánto tiempo después de iniciar cada terapia modificadora de la enfermedad se observa el mayor efecto y su estabilización. A este fenómeno lo denominaron "retraso terapéutico" ^22^ y, aunque es prometedor, requiere una mayor validación externa.

Con base en la evidencia disponible sobre los tiempos de inicio de acción de las terapias modificadoras de la enfermedad y el momento ideal para practicar una nueva RM, que será el examen de referencia para comparar las imágenes subsecuentes, se recomiendan los siguiente tiempos ^23^:

- Para las terapias modificadoras de la enfermedad de reconstitución inmunológica (cladribina y alemtuzumab), se considera un año después de la segunda dosis, o sea, a los dos años de iniciar la terapia.
- Para el acetato de glatiramer, se consideran nueve meses.
- Para los demás medicamentos, se consideran seis meses.

La recomendación basada en la evidencia científica, con un tiempo discordante para el acetato de glatiramer, sugiere que el medicamento alcanza su máximo efecto sobre los resultados radiológicos después de siete meses ^23^. Estos tiempos deben utilizarse como guía para la correcta definición de falla terapéutica en cada caso individual.

## ¿Cómo debe ser el seguimiento de un paciente con un síndrome radiológico aislado?

El síndrome radiológico aislado consiste en la detección incidental de imágenes indicativas de una enfermedad desmielinizante del tipo de la esclerosis múltiple en un paciente sin signos ni síntomas de esta enfermedad. Su diagnóstico se basa principalmente en la interpretación de la RM, considerando la más reciente revisión de los criterios de McDonald.

Una vez se presenta un evento neurológico clínico en un paciente con síndrome radiológico aislado, se pueden aplicar los criterios diagnósticos para soportar un diagnóstico definitivo de esclerosis múltiple. La historia natural del síndrome radiológico aislado y sus implicaciones en la práctica clínica, son objeto de debate constante en el campo de las enfermedades desmielinizantes ^24^.

Okuda, *et al.,* en el 2014, llevaron a cabo un estudio retrospectivo y evaluaron 451 sujetos con síndrome radiológico aislado obtenidos a partir de 22 bases de datos de cinco países. Hicieron el seguimiento desde la RM inicial hasta el primer evento clínico relacionado con desmielinización y calcularon el riesgo de que estos pacientes desarrollaran un evento clínico agudo o progresivo sugestivo de esclerosis múltiple a los cinco años.

Se detectaron eventos clínicos en 34 % (error estándar = 3 %) de los individuos dentro de un período de cinco años a partir del primer estudio de RM. En el modelo multivariado, la edad "menor de 37 años", con un cociente de riesgos instantáneos *(Hazard Rate,* HR) de 0,98 (IC_95%_ 0,96-0,99; p= 0,03), el sexo "hombre, con un HR de 1,93 (IC_95%_ 1,24-2,99; p=0°,004) y la presencia de lesiones en la médula espinal cervical o torácica, con un HR de 3,08 (IC_95%_ 2,06-4,62; p=0,001), se identificaron como factores predictores significativos para el desarrollo de un primer evento clínico y un mayor riesgo de desarrollar esclerosis múltiple ^25^.

Actualmente, con base en los hallazgos de este estudio, la vigilancia activa, clínica e imagenológica recomendada para estos pacientes, se debe hacer a los 6 y 12 meses en el primer año después del diagnóstico y, anualmente, durante cinco años ^24^^,^^25^.

Más recientemente, Lebrun, *et al.,* realizaron un seguimiento extendido a 10 años sobre la misma población estudiada por Okuda en el 2014. Concluyeron que el riesgo de conversión de un síndrome radiológico aislado a una esclerosis múltiple aumentaba con el tiempo, siendo del 51,2 % a 10 años.

Los factores independientes relacionados con un mayor riesgo de progresión del síndrome a esclerosis múltiple fueron: tener una menor edad en el momento del diagnóstico del síndrome radiológico aislado, un líquido cefalorraquídeo con bandas oligoclonales positivas, y lesiones infratentoriales o en la médula espinal. La actividad radiológica durante el seguimiento de estos pacientes también se asoció con un mayor riesgo de presentar un evento clínico (HR=1,81) (IC_95%_ 1,31-2,52; p<0,001). A diferencia de los hallazgos previamente reportados a los cinco años, el sexo masculino ya no se consideró como un factor pronóstico para la evolución clínica, con un HR de 1,24 (IC_95%_ 0,88-1,77; p=0,25).

A la luz de estos hallazgos, se podría afirmar que el seguimiento clínico e imagenológico de los pacientes con síndrome radiológico aislado a futuro debería extenderse a 10 años ^26^.

## Los pacientes con síndrome clínico aislado que no cumplen los criterios para esclerosis múltiple, ¿deben recibir terapias modificadoras de la enfermedad?

Un síndrome clínico aislado sugestivo de esclerosis múltiple es el primer episodio clínico monofásico en el que el paciente refiere síntomas típicos de esclerosis múltiple (por ejemplo, neuritis óptica unilateral, mielitis transversa, síndrome cerebeloso o síndrome de tallo). Hay hallazgos objetivos focales o multifocales que reflejan un evento desmielinizante en el sistema nervioso central ^27^. Al igual que una recaída típica de esclerosis múltiple, este episodio debe durar al menos 24 horas, con recuperación o sin ella, descartando la presencia de fiebre o infección. En este caso, si bien el paciente presenta un síntoma o signo típico de esclerosis múltiple, aún no cumple los criterios diagnósticos de McDonald de 2017 ^27^.

Los estudios controlados contra placebo, en que se ha evaluado el resultado en los pacientes con un primer evento desmielinizante y que han recibido terapias modificadoras de la enfermedad, sin cumplir los criterios para esclerosis múltiple, son:

1. CHAMPS (Interferón beta 1 a 30 μg una vez por semana)
2. ETOMS (Interferón beta 1 a 22 μg una vez por semana)
3. BENEFIT (Interferón subcutáneo beta 1 b 250 μg cada 24 horas)
4. REFLEX (Interferón beta 1 a 44 μg 3 veces por semana)
5. PreCISE (Acetato de glatiramer 20 μg una vez al día)
6. ORACLE (Cladribine)
7. 7 TOPIC (Teriflunamida)

Estos estudios, en general, incluyeron adultos (>18 años hasta los 55 años) quienes, además de tener un síndrome clínico aislado, debían tener alguna lesión inflamatoria sugestiva de esclerosis múltiple en el sistema nervioso central evidenciada por RM cerebral. Los resultados muestran de forma constante que el inicio temprano de estos terapias modificadoras de la enfermedad retrasa la conversión a esclerosis múltiple definitiva ^28^^-^^34^.

Por ello, los pacientes entre los 18 y los 55 años con un síndrome clínico aislado típico de esclerosis, que no cumplan a cabalidad los criterios de McDonald de 2017, pero que presenten en la RM lesiones típicas de esclerosis múltiple, podrían ser candidatos para iniciar una terapia modificadora de la enfermedad. Su elección debe ser individualizada para cada paciente.

Finalmente, es importante aclarar que, en los estudios mencionados previamente, no se utilizaron los criterios diagnósticos actuales de esclerosis múltiple (McDonald, 2017) ^27^. Es de resaltar que se desconoce si, al usar los criterios actuales en esta población con síndrome clínico aislado, se puede confirmar un diagnóstico de esclerosis múltiple en algunos de ellos ^27^. Se requieren estudios aleatorizados controlados en que se utilicen los criterios actuales de McDonald y que incluyan seguimiento a largo plazo.

## ¿Puede la administración de natalizumab, en dosis extendidas, reducir el riesgo de leucoencefalopatía multifocal progresiva en pacientes con esclerosis múltiple?

En la actualidad, el natalizumab es uno de los tratamientos de gran eficacia más usados en la esclerosis múltiple. Actúa como inhibidor de la integrina alfa 4, evitando el ingreso de células T inflamatorias al sistema nervioso central.

En el año 2004, fue aprobado por primera vez por la *Food and Drug Administration* (FDA) para la esclerosis múltiple ^35^. Posteriormente, en 2005, fue retirado del mercado a raíz de dos casos de pacientes que desarrollaron leucoencefalopatía multifocal progresiva, otra enfermedad desmielinizante causada por infección por el virus John Cunningham (JCV) en las células del sistema nervioso central. Finalmente, en 2006, fue reintroducido bajo un programa de prevención y mitigación de riesgos llamado *Tysabri Outreach Unified Commitment to Health* (TOUCH) ^36^.

A partir de la base de datos de este programa, se han podido aclarar los factores de riesgo relacionados con el desarrollo de la leucoencefalopatía multifocal progresiva. Los principales tres son:

1. tratamiento por más de 24 meses ^37^,
2. uso previo de inmunosupresores, y
3. exposición previa al virus JC (anticuerpos anti-JCV positivos) ^38^.

Recientemente, se ha venido investigando si el disminuir la frecuencia de la infusión intravenosa del medicamento podría reducir el riesgo de leucoencefalopatía multifocal progresiva. Esta forma de dosificar el fármaco se ha denominado dosis de intervalo extendido, la cual consiste en administrar las infusiones separadas por periodos de más de cuatro semanas, que es el tiempo establecido en la dosis de intervalo estándar.

La administración de las dosis en periodos estándar lleva a que se saturen más del 80 % de los receptores de integrina alfa 4 después de un mes de su administración ^39^. Se ha propuesto que una dosis en un rango intermedio, que vaya de 4 a 10 semanas, podría resultar en una reducción en la concentración valle del medicamento (al no posponer tanto su administración) y, a su vez, en una reducción de la cantidad de receptores de integrina saturados, con el fin de que siga existiendo algo de transmigración celular que evite la entrada del JCV al sistema nervioso central ^40^.

En 2014, se publicó una revisión retrospectiva de 361 pacientes que recibieron natalizumab durante, por lo menos, seis meses. De estos pacientes, 96 lo recibieron con intervalos extendidos, cada 6 a 8 semanas, en algún momento de su tratamiento. Se encontró que la tasa de recaídas de la enfermedad no fue diferente entre los dos grupos (con intervalo extendido y con intervalo estándar), por lo cual se concluyó que el extender el intervalo de las dosis no compromete de forma estadísticamente significativa la efectividad del tratamiento ^39^.

De manera similar, en otra revisión retrospectiva publicada en 2016, se incluyeron 2.004 pacientes, de los cuales 905 recibieron las dosis con intervalos extendidos, hasta cada 8 semanas. Se encontró que no hubo diferencias significativas entre los grupos según el intervalo de las dosis, en cuanto a la ausencia de actividad (clínica y radiológica) de la enfermedad y la tasa anual de recaídas.

En cuanto a casos de leucoencefalopatía multifocal progresiva, la cantidad de pacientes fue muy pequeña para aclarar este punto con un poder estadístico suficiente. Sin embargo, es de resaltar que en el grupo con dosis en intervalos estándar, hubo 4 casos de leucoencefalopatía multifocal progresiva, mientras que, en el de intervalos extendidos, no hubo ningún caso ^41^.

Solo en aquel momento se pudo establecer, con la información disponible, que las dosis en intervalos extendidos no alteraban la eficacia del tratamiento con natalizumab; sin embargo, persistía la duda de si el extender los intervalos podría reducir el riesgo de leucoencefalopatía multifocal progresiva.

Posteriormente, en 2019, se publicó un estudio de cohorte retrospectivo, basado en la base de datos TOUCH, cuyo objetivo principal era evaluar si las dosis de intervalo extendido podían reducir el riesgo de leucoencefalopatía multifocal progresiva en pacientes con factores de riesgo. Este estudio incluyó un total de 35.521 pacientes positivos para anticuerpos anti-JCV y con historia de uso previo de inmunosupresores.

En el análisis primario, hubo una reducción del 94 % en el riesgo de leucoencefalopatía multifocal progresiva en los últimos 18 meses de tratamiento al extender los intervalos, en comparación con las dosis en intervalos estándar, y una diferencia estadísticamente significativa en el cociente de riesgos instantáneos o *hazards rates* (HR), el cual fue menor con la extensión de los intervalos.

En el análisis secundario, hubo una reducción del 88 % del riesgo de leucoencefalopatía multifocal progresiva en cualquier momento durante el tratamiento, con la dosis de intervalo extendido en comparación con el estándar, y una diferencia estadísticamente significativa en el el cociente de riesgos instantáneos (HR), cuyo valor fue menor con la extensión del intervalo.

En el análisis terciario se evaluaron 815 pacientes que habían recibido dosis de intervalo extendido en algún momento a lo largo de toda su historia de tratamiento, comparándolos con los que solo recibieron dosis de intervalo estándar, y ninguno presentó leucoencefalopatía multifocal progresiva.

Los resultados de los tres análisis mostraron reducción del riesgo de desarrollar esta leucoencefalopatía con los intervalos extendidos, con valores clínica y estadísticamente significativos ^42^.

El análisis de la base de datos TOUCH se repitió en 2018, agregándole 4.888 nuevos pacientes con un aumento relativo del número de pacientes con dosis de intervalo extendido, en comparación con aquellos con intervalo estándar. Se siguieron encontrando diferencias significativas en el riesgo relativo (RR) en el análisis primario y el secundario (disminución de 94 % y 88 %, respectivamente) y, en el terciario, siguieron sin aparecer casos de leucoencefalopatía multifocal progresiva.

Además, los factores de riesgo para desarrollar la leucoencefalopatía siguieron siendo los mismos que con las dosis de intervalo estándar, pues la mayoría de los casos que ocurrieron con dosis de intervalo extendido tenían un índice de JCV mayor o igual a 1,5, o exposición previa a inmunosupresores. En conclusión, el nuevo análisis corroboró los datos del original y que ambos tipos de dosis, con intervalo extendido o estándar, comparten los mismos factores de riesgo para desarrollar leucoencefalopatía multifocal progresiva ^42^.

Los datos indican que el riesgo de leucoencefalopatía se reduce al administrar las dosis de natalizumab en intervalos extendidos. Sin embargo, se requiere un seguimiento en el tiempo con análisis periódicos de los datos del TOUCH, para asegurarse de que, efectivamente, sí lo reduce y no solo lo retrasa. La información actualmente disponible es puramente observacional y, por lo tanto, no se ha emitido una recomendación formal al respecto; sin embargo, se encuentra en curso una prueba clínica aleatorizada en fase 3, denominada NOVA ^43^.

## ¿Se deben tratar las recaídas leves de esclerosis múltiple?

Actualmente, no hay una definición ampliamente aceptada sobre qué es una recaída leve de esclerosis múltiple. Las definiciones actuales hacen referencia a la afectación de un solo sistema (idealmente, sin compromiso motor o cerebeloso), la cual no compromete la capacidad de llevar a cabo de forma independiente las actividades de la vida diaria y tiende a recuperarse de forma completa ^44^.

Las recaídas con síntomas visuales, sensitivos y de tallo, suelen recuperarse de forma más completa que cuando se presentan síntomas cerebelosos, motores y de esfínteres, los cuales suelen causar más secuelas a largo plazo ^45^. También, se ha utilizado una definición basada en el cambio en el puntaje EDSS durante la crisis como un marcador de gravedad, dado que las recaídas que cambian en 1 o menos puntos el EDSS respecto al basal, producen menor riesgo de discapacidad a largo plazo ^46^.

La información disponible ha demostrado de forma constante que se logra una recuperación más rápida del déficit neurológico, cuando se incluyen bolos de metilprednisolona durante el tratamiento de una recaída de la enfermedad ^47^. Sin embargo, este manejo no ha demostrado prevenir recaídas o cambiar la acumulación de discapacidad a largo plazo ^48^.

Aunque los bolos de metilprednisolona no han demostrado tener un impacto sobre los resultados clínicos a largo plazo, se ha observado mediante imágenes avanzadas *(Magnetization Transfer Ratios)* que producen una mayor recuperación del tejido neuronal, la cual se mantiene hasta por 19 meses ^49^. Además, es importante tener en cuenta los resultados de importancia para el paciente, pues las recaídas también producen carga emocional, psicológica, económica y familiar, entre otras ^46^.

Por último, como para toda decisión clínica, es fundamental analizar los riesgos, costos y beneficios. En general, los bolos de corticoides en esquemas cortos, como se utilizan en las recaídas de la esclerosis múltiple, no se consideran muy costosos, no se asocian con mayores efectos secundarios, y han demostrado beneficios clínicos y radiológicos; estos están mejor demostrados a corto plazo, pero también, se ha encontrado que persisten a mediano y probablemente a largo plazo.

Por esta razón, se recomienda tener un umbral bajo para el tratamiento de las recaídas leves de esclerosis múltiple. No obstante, cuando las recaídas se consideran clínicamente muy leves, se podría considerar omitir su tratamiento inmediato. La disponibilidad de tratamiento oral o intravenoso domiciliario, inclinaría aún más la balanza hacia tratar hasta las recaídas más leves de esclerosis múltiple.

## Los pacientes que reciben pulsos de metilprednisolona intravenosa, ¿requieren tratamiento preventivo para evitar la hiperinfección por *Strongyloides stercoralis?*

La infección por *Strongyloides stercoralis* es frecuente en regiones del trópico y subtrópico. Colombia se considera un área endémica, con una prevalencia reportada de 16 a 19 % ^50^.

La larva filiforme de S. *stercoralis* que se encuentra en los suelos, ingresa a través de la piel, alcanza los alvéolos por vía sanguínea, asciende por la vía respiratoria y es deglutida; ya en el intestino delgado, madura y deposita sus huevos. Posteriormente, se liberan las larvas rabditiformes en las heces y, de esta manera, los sujetos se pueden reinfectar si las larvas penetran la piel perianal, lo cual ocurre con mayor frecuencia en sujetos inmunosuprimidos.

En personas inmunocompetentes, puede causar infecciones asintomáticas crónicas durante décadas. Sin embargo, los pacientes inmunosuprimidos tienen el riesgo de presentar una hiperinfección con diseminación a pulmón, hígado y cerebro. La hiperinfección produce, además, una sepsis sistémica secundaria a la translocación de bacterias del tubo digestivo causada por la invasión de la larva en la pared intestinal. Este cuadro clínico se acompaña de gran mortalidad, la cual varía entre 85 y 100 % ^51^.

En efecto, la hiperinfección puede ocurrir con grandes o pequeñas dosis de esteroides, incluso con su aplicación local. Se ha descrito en pacientes que han recibido dexametasona a dosis de 1 mg/día durante ocho semanas y con cursos cortos de prednisolona durante 6 a 17 días ^52^^,^^53^. A la fecha, no se han reportado casos de hiperinfección por *S. stercoralis* en pacientes que hayan recibido pulsos de metilprednisolona para tratar las recaídas de esclerosis múltiple.

Idealmente, antes de formular esteroides, se debería averiguar si el paciente tiene una infección crónica por S. *stercoralis,* con el fin de erradicar el nematodo y prevenir la hiperinfección. Sin embargo, el reconocer sujetos con infección crónica es un reto diagnóstico, pues la mayoría son asintomáticos clínicamente. Además, la sintomatología puede ser inespecífica y consistir en vómito intermitente, diarrea, estreñimiento, borborigmos, prurito e hiperreactividad bronquial ^51^.

La prueba serológica parece ser el examen más sensible en áreas endémicas, pero las pruebas de laboratorio son costosas y sus resultados pueden tardar hasta dos semanas. Por esto, algunos autores recomiendan el tratamiento empírico para erradicar al nematodo en pacientes con grandes riesgos, como vivir en áreas endémicas, tener un bajo nivel socioeconómico, consumir alcohol, trabajar en el campo o estar expuesto por la minería de carbón.

Como Colombia se considera un área endémica para *S. stercoralis,* antes de administrar esteroides es importante analizar cada caso en particular y, según los factores de riesgo y el criterio médico, decidir si se usa empíricamente un antiparasitario contra el nematodo. Uno de los esquemas terapéuticos más recomendados es el de 400 mg de albendazol cada 12 horas durante tres días; asimismo, se pueden utilizar la ivermectina y el tiabendazol ^54^.

## ¿Las vacunas aumentan el riesgo de enfermedades desmielinizantes tales como la encefalomielitis aguda diseminada, la esclerosis múltiple y el síndrome de Guillain-Barré?

La primera manifestación neurológica asociada con la vacunación fue el síndrome neuroparalítico posterior a la inmunización contra la rabia en el año 1889. Desde entonces, las vacunas se han asociado con encefalitis, síndrome de Guillain-Barré, crisis convulsivas, cefalea, neuropatía y otros síndromes neurológicos ^55^.

La asociación entre el síndrome de Guillain-Barré y la vacunación data del año 1976 durante la inmunización contra la influenza porcina en Estados Unidos. En este periodo de vacunación, se identificó un riesgo de un caso por cada 100.000 pacientes vacunados ^56^. Asimismo, en un metanálisis publicado en 2015, se concluyó que existe un riesgo de 1,41 de que se presente un síndrome de Guillain-Barré después de la vacunación contra influenza. Sin embargo, en un estudio anglosajón no se encontró asociación en los primeros 90 días después de la inmunización (RI=0,76) ^57^.

En general, se considera que el riesgo de síndrome de Guillain-Barré por la vacuna de influenza es de 1 a 2 casos extra por cada millón de personas vacunadas. Esta pequeña contingencia no supera los beneficios de la vacunación y, además, el riesgo de desarrollar un síndrome de Guillain-Barré es 4 a 7 veces mayor que el asociado con la vacunación. Otras vacunas, como aquellas contra sarampión, paperas, rubéola, conjugado meningocócico, poliomielitis, neumococo, varicela, *Haemophilus influenzae* de tipo B, rabia, tétanos, difteria, hepatitis A y hepatitis B, no incrementan el riesgo de sufrir este síndrome ^57^.

Por otra parte, la incidencia posvacunal de encefalomielitis aguda diseminada es de 0,1 a 0,2 por cada 100.000, y el riesgo parece ser mayor con la vacuna contra el sarampión ^58^. En un estudio poscomercialización en Japón, entre 1994 y 2004, se documentaron 7 casos de encefalomielitis aguda diseminada por cada 67,2 millón de vacunas ^59^. Sutton, *et al.,* describieron cinco casos de encefalomielitis aguda diseminada después de la dosis de refuerzo de la vacuna contra el virus del papiloma humano (HPV) ^60^.

Por el contrario, en un estudio realizado en Dinamarca y Suecia, no se encontró una relación causal entre la vacuna contra el HPV y la aparición de enfermedades desmielinizantes ^61^. Asimismo, en otro estudio de casos y controles entre 2011 y 2015, no se encontró aumento en el riesgo de encefalomielitis aguda diseminada en adultos vacunados contra hepatitis B, influenza, poliomielitis, difteria, tétano, paperas, sarampión, rubéola, hepatitis A, varicela o rabia ^62^.

En Francia, en el año 1990, se documentaron casos de esclerosis múltiple después de la vacunación contra la hepatitis B ^62^. Sin embargo, en estudios más recientes, no se ha encontrado una asociación causal y la revisión sistémica de la literatura lo confirma con un OR de 0,965 de esclerosis múltiple posterior a la vacunación contra la hepatitis B ^62^. En otros estudios, se ha encontrado que las vacunas contra HPV, influenza, tétanos, difteria, tosferina, poliomielitis y fiebre tifoidea, y la triple viral, no aumentan el riesgo de esclerosis múltiple ^61^^,^^62^.

Respecto a la vacuna contra la fiebre amarilla, en un estudio se reportó que cinco de siete pacientes con esclerosis múltiple vacunados desarrollaron una recaída después de la vacunación ^63^. Sin embargo, más recientemente en un estudio retrospectivo, se demostró que la vacunación no se asoció con mayores tasas de recaídas, ni con nuevas lesiones inflamatorias evidenciadas por RM ^64^.

En conclusión, la vacuna contra la influenza ha demostrado tener muy poco riesgo de inducir un síndrome de Guillain-Barré, que no supera sus beneficios a nivel poblacional. No existe información suficiente para confirmar que la vacunación aumente el riesgo de encefalomielitis aguda diseminada en adultos. Las vacunas contra hepatitis B, HPV, influenza, tétanos, difteria, tosferina, poliomielitis y fiebre tifoidea, y la triple viral, no aumentan el riesgo de esclerosis múltiple.

Respecto a la vacuna contra la fiebre amarilla y las recaídas de esclerosis múltiple, hay datos contradictorios y es necesario sumar nuevas investigaciones en este campo.

## ¿Cuándo suspender la terapia modificadora de la enfermedad en pacientes con esclerosis múltiple?

El papel de las terapias modificadoras de la enfermedad en la esclerosis múltiple es modular o disminuir la actividad del sistema inmunológico, con el fin de atenuar la inflamación que afecta el sistema nervioso central. Constantemente, el beneficio de estas ha demostrado ser mayor en pacientes con enfermedad inflamatoria activa ^65^^,^^66^. Históricamente, se han cuestionado las terapias modificadoras de la enfermedad en pacientes de mayor edad, no sólo por aspectos de seguridad, sino también, por la historia natural de la esclerosis múltiple, cuyo riesgo de nuevos episodios de inflamación tiende a ser menor con el paso de los años ^67^. En efecto, se ha visto que a partir de los 50 años, la tasa anual de recaídas es inferior al 10 % ^66^. Este fenómeno podría ser secundario a la inmunosenescencia y convoca a la discusión sobre la posibilidad de suspender la terapia modificadora de la enfermedad en algunos pacientes con esclerosis múltiple.

Los resultados de un metaanálisis reciente sugieren que la mitad de los sujetos menores de 53 años con terapias modificadoras de la enfermedad, están expuestos a efectos adversos acumulativos, con poco o ningún beneficio terapéutico ^68^. Además, los datos provenientes de *MSBase* han demostrado que los sujetos más jóvenes con menor discapacidad y con lesiones en la RM realzadas con el medio de contraste en los últimos tres años, son aquellos que tienen un mayor riesgo de recurrencia de recaídas al suspender la terapia modificadora de la enfermedad ^66^. Actualmente, con el estudio DISCOMS, que se encuentra en curso, se busca evaluar la seguridad de suspender la terapia modificadora de la enfermedad en sujetos con diagnóstico de esclerosis múltiple de tipo recaída-remisión, que no hayan presentado recaídas en los últimos cinco años y que no tengan nuevas lesiones evidenciadas por RM en los últimos tres años ^69^.

La suspensión de la terapia modificadora de la enfermedad podría considerarse en pacientes de mayor edad (probablemente, mayores de 53 años), con una enfermedad quiescente clínica y radiológicamente por periodos significativos de cuatro años, por lo menos, y cuando los riesgos superen los beneficios del tratamiento. Los resultados del estudio DISCOMS permitirán tener una información más sólida para soportar o refutar esta consideración. De forma similar, en pacientes con formas progresivas de la enfermedad y sin signos de inflamación, podría considerarse retirar la terapia modificadora de la enfermedad cuando los riesgos superen los beneficios.

## ¿Cuál es el conocimiento actual sobre un segundo ciclo de inmunoglobulina intravenosa en pacientes con síndrome de Guillain-Barré?

El síndrome de Guillain-Barré es una de las causas más frecuentes de ingreso a la unidad de cuidados intensivos, por debilidad muscular y parálisis aguda. La inmunoglobulina (Ig) intravenosa se considera efectiva para el tratamiento temprano del síndrome de Guillain-Barré. El esquema estándar de Ig intravenosa (2 g/kg en 2 a 5 días) acelera el tiempo de recuperación si se administra dentro de las primeras dos semanas después de iniciarse el cuadro clínico ^70^^,^^71^. A pesar del tratamiento inmunomodulador, la evolución clínica es desfavorable en algunos pacientes y se ha interrogado la necesidad de otras aproximaciones terapéuticas ^72^, incluyendo un segundo esquema de inmunoglobulina intravenosa ^71^.

Verboon, *et al.,* realizaron un estudio observacional, prospectivo y multicéntrico, para evaluar la efectividad de un segundo esquema de inmunoglobulina intravenosa. En este estudio, se tuvo en cuenta el estudio internacional de pronóstico del síndrome de Guillain-Barré *(International GBS Outcome Study,* IGOS) y se seleccionaron sujetos cuyo puntaje en la escala modificada de pronóstico EGOS *(Erasmus GBS Outcome Score)* en la primera semana, predijera un pronóstico desfavorable.

Los pacientes que recibieron un segundo esquema de inmunoglobulina intravenosa se dividieron en dos grupos: en el primero, aquellos que lo habían recibido de forma temprana (antes de dos semanas), y en el segundo, aquellos con administración tardía (2 a 4 semanas).

En el estudio se evaluaron los resultados primarios y secundarios en ambos grupos, comparándolos con un grupo control. Entre los primarios, se evaluó el resultado funcional de la escala de discapacidad del síndrome de Guillain-Barré. Entre los resultados secundarios, se incluyeron: la capacidad de caminar de forma independiente a las 26 semanas; la necesidad de asistencia respiratoria mecánica durante cualquier momento del seguimiento; el tiempo de estancia en la unidad de cuidado intensivo; el tiempo de necesidad de soporte respiratorio; la mortalidad relacionada con el síndrome de Guillain-Barré a los 6 meses; las fluctuaciones relacionadas con el tratamiento, y otras complicaciones.

No se encontraron diferencias estadísticamente significativas entre los grupos, para los resultados estudiados. Aunque en el análisis se incluyó un grupo reducido de pacientes, este estudio refleja la práctica clínica actual en pacientes con síndrome de Guillain-Barré con un pronóstico desfavorable; un segundo esquema de inmunoglobulina intravenosa no mostró ningún efecto positivo en su resultado funcional ^72^.

Recientemente, en el estudio clínico doble ciego, aleatorizado y controlado con placebo, titulado SID-GBS y publicado por Walgaard, *et al.,* se evaluó nuevamente el beneficio del segundo esquema de inmunoglobulina intravenosa (2 g/kg por 5 días). Se incluyeron 327 pacientes con diagnóstico de síndrome de Guillain-Barré, de los cuales 112 tenían un pronóstico desfavorable según la puntuación para discapacidad Erasmus. El análisis por intención de tratar incluyó 93 pacientes con mal pronóstico: 49 (53 %) recibieron un segundo esquema de inmunoglobulina intravenosa y 44 (47 %) recibieron placebo.

Los resultados demostraron que este segundo esquema no era superior al placebo, con una razón de probabilidades común ajustada para mejorar la discapacidad a las cuatro semanas de 1,4 (IC_95%_ 0,6-3,3; p=0,45). Además, los pacientes que recibieron el segundo esquema presentaron más reacciones adversas en los primeros 30 días después del tratamiento (35 Vs. 16 %), incluyendo eventos tromboembólicos. Por lo tanto, este estudio reafirma que los pacientes con síndrome de Guillain-Barré y con criterios de mal pronóstico, no se benefician de esta intervención, la cual puede condicionar una mayor tasa de eventos adversos ^73^.

Actualmente, solo se considera administrar un segundo esquema de inmunoglobulina intravenosa en aquellos pacientes con síndrome de Guillain-Barré que cursan con fluctuaciones relacionadas con el tratamiento ^74^^,^^75^.

## ¿Como diferenciar el síndrome de Guillain-Barré de una polineuropatía crónica inflamatoria desmielinizante de inicio agudo?

Generalmente, los pacientes con síndrome de Guillain-Barré tienen un curso clínico monofásico con un nadir a las 4 semanas, a diferencia de los sujetos con polineuropatía crónica inflamatoria desmielinizante, quienes presentan un curso clínico progresivo en 8 semanas. Sin embargo, el 5 % de aquellos que padecen el síndrome de Guillain-Barré pueden progresar a una polineuropatía crónica inflamatoria desmielinizante y, de estos últimos, el 16 % pueden hacerlo de forma aguda ^70^.

Aunque diferenciar un síndrome de Guillain-Barré de una polineuropatía crónica inflamatoria desmielinizante de inicio agudo puede ser un reto diagnóstico complejo, es de vital importancia pues estas enfermedades tienen diferentes implicaciones terapéuticas y pronósticas.

Los resultados de estudios de series de casos y de estudios retrospectivos, indican que los signos sensitivos, como ataxia e hipopalestesia, se presentan con mayor frecuencia en pacientes con sospecha diagnóstica de polineuropatía crónica inflamatoria desmielinizante de inicio agudo o síndrome de Guillain-Barré. Además, en los pacientes con esta polineuropatía de inicio agudo es poco frecuente documentar disautonomías o debilidad facial y, además, por lo general no requieren asistencia respiratoria mecánica ^76^^,^^77^. El pródromo infeccioso y el compromiso motor más grave ocurren con mayor frecuencia en los pacientes con el síndrome de Guillain-Barré ^76^.

La evolución de la enfermedad es un elemento clave para hacer el diagnóstico diferencial. Si el paciente tiene tres o más fluctuaciones relacionadas con el tratamiento, o presenta deterioro después de la octava semana, es más probable que se trate de una polineuropatía crónica inflamatoria desmielinizante de inicio agudo ^78^. Los hallazgos tempranos de electromiografía y neuroconducción, así como el estudio de líquido cefalorraquídeo, no permiten diferenciar de forma confiable estas dos enfermedades^76^^-^^78^.

## ¿Cuál es el rol de la enzima convertidora de angiotensina en líquido cefalorraquídeo para el diagnóstico de neurosarcoidosis?

La sarcoidosis es una enfermedad granulomatosa sistémica, que puede llegar a comprometer el sistema nervioso central en 5 a 25 % de los casos ^79^ . Su presentación clínica variable y su curso heterogéneo frecuentemente dificultan y retrasan su diagnóstico. Actualmente, el diagnóstico de neurosarcoidosis se clasifica de acuerdo con el grado de certeza, en *posible, probable* o *definitivo.* Es de mencionar que, en el contexto particular de la confirmación diagnóstica definitiva, la cual requiere de una biopsia de tejido nervioso, surge la necesidad de descubrir un biomarcador menos invasivo y con mejor rendimiento.

La enzima convertidora de angiotensina (ECA) es una glucoproteína de membrana producida por las células epitelioides del tejido granulomatoso, que cumple un rol esencial en la fisiopatología de la sarcoidosis. La ECA se eleva en suero en 30 a 80 % de los casos, con una sensibilidad y especificidad variables entre 22 y 86 % y entre 54 y 95 %, respectivamente ^80^ . A la fecha, la información sobre su utilidad en el diagnóstico de la neurosarcoidosis se basa, principalmente, en estudios observacionales.

En 1985, se publicó en *Neurology* la primera serie retrospectiva de casos, en que se estudió el aumento de la ECA en el líquido cefalorraquídeo de pacientes con neurosarcoidosis. Se encontró que, de 20 pacientes con neurosarcoidosis, 11 (55 %) presentaban niveles elevados de la ECA, en comparación con 1 de 12 pacientes con sarcoidosis sistémica sin neurosarcoidosis. A pesar de las limitaciones del estudio y el bajo poder estadístico, se concluyó que los niveles de la ECA en el líquido cefalorraquídeo podían ser potencialmente útiles en el diagnóstico de neurosarcoidosis ^81^.

Posteriormente, en 1999, Dale, *et al.,* publicaron una revisión retrospectiva de 110 pacientes, en quienes se cuantificó la ECA en el líquido cefalorraquídeo para descartar neurosarcoidosis. Encontraron que el 56 % de pacientes con aumento de la ECA no cursó con neurosarcoidosis y, a su vez, que el 76 % de aquellos con diagnóstico de neurosarcoidosis tenían valores normales , cuestionándose así la utilidad clínica de este biomarcador ^82^.

Más recientemente, Tahmoush, *et al.,* publicaron una serie retrospectiva de 207 casos. Realizaron la medición de la ECA en el líquido cefalorraquídeo de 11 pacientes que cumplían con los criterios para un diagnóstico probable de neurosarcoidosis y los compararon con 207 controles. Encontraron que, por radioensayo, la actividad promedio de la ECA en el líquido cefalorraquídeo era significativamente mayor (p<0,05) en los 11 casos de probable neurosarcoidosis (9,5 ± 6,9 nmol/ml/minuto), en comparación con el grupo control (2,9 ± 2,7 nmol/ml/minuto). También, encontraron que el punto de corte óptimo discriminador de actividad de ECA en el líquido cefalorraquídeo fue de 8 nmol/ml/minuto, para una sensibilidad del 55 % y una especificidad del 94 %. Se concluyó, entonces, que la ECA en líquido cefalorraquídeo podría ser un biomarcador útil para el diagnóstico de neurosarcoidosis probable, en especial, cuando hay lesiones que se realzan en la neuroimagen ^83^.

Teniendo en cuenta la evidencia hasta el año 2009, Khoury, *et al.,* publicaron una evaluación del tema de interés con base en los dos artículos primarios ^82^^,^^83^. Concluyeron que la prueba de ECA en líquido cefalorraquídeo tiene una baja sensibilidad (24-55 %), pero, que es razonablemente específica (94-95 %) para el diagnóstico de neurosarcoidosis, pudiéndose utilizar para aumentar la probabilidad pretest. Sin embargo, también concluyeron que su precisión diagnóstica no está claramente establecida y no supera la del diagnóstico histopatológico ^84^.

Más adelante, en 2015, Bridel, *et al.,* llevaron a cabo un estudio retrospectivo que incluyó 440 pacientes sometidos a punción lumbar por sospecha diagnóstica de neurosarcoidosis. Del total de sujetos estudiados, 8 cumplieron con los criterios para neurosarcoidosis probable y, uno, para neurosarcoidosis definitiva. Se encontró que no hubo diferencias estadísticamente significativas en los valores de la ECA en líquido cefalorraquídeo entre los 9 pacientes con diagnóstico de neurosarcoidosis (media=4,3 y desviación estándar=4,7) y los controles (media=2,3 y desviación estándar=2,7) (p=0,11). A su vez, los valores séricos de ECA fueron significativamente más altos en los 9 pacientes con neurosarcoidosis (media=77,2 y desviación estándar=4,9,5) que en los controles (media=32,8 y desviación estándar=2,7) (p=0,11).

Además, trazaron las curvas ROC para determinar el punto de corte de la ECA en líquido cefalorraquídeo como prueba diagnóstica, y encontraron el corte de mayor discriminación en 2, para una sensibilidad y especificidad de apenas 66,7 % y 67,3 %, respectivamente; sin tampoco vislumbrar una buena razón de verosimilitud ^85^.

Hasta el momento, la información disponible está basada en pequeñas series retrospectivas de casos y la mayoría incluyen controles con comparadores sanos. El estudio con el mayor número de pacientes es el publicado por Bridel, e*t al.*^85^, en el cual los controles fueron pacientes clínicamente sospechosos de neurosarcoidosis, lo cual lo convierte en el más similar a los casos clínicos reales.

Los resultados entre estudios son contradictorios, pero, en su mayoría, indican que la ECA en líquido cefalorraquídeo tiene poca sensibilidad y una especificidad muy variable, y su rendimiento diagnóstico es pobre debido a una pequeña razón de verosimilitud y a una pequeña área bajo la curva ROC. Por lo tanto, es una prueba de poco valor diagnóstico y no reemplaza la biopsia, y la información actual es insuficiente para recomendar su uso clínico.

## References

[1] Optic Neuritis Treatment Trial Headquarters, University of South Florida College of Medicine (1991). The clinical profile of optic neuritis. Arch Ophthalmol.

[2] Perumal JS, Caon C, Hreha S, Zabad R, Tselis A, Lisak R (2008). Oral prednisone taper following intravenous steroids fails to improve disability or recovery from relapses in multiple sclerosis. Eur J Neurol.

[3] Lević Z, Micić D, Nikolić J, Stojisavljević N, Sokić D, Janković S (1996). Short-term high dose steroid therapy does not affect the hypothalamic-pituitary-adrenal axis in relapsing multiple sclerosis patients. Clinical assessment by the insulin tolerance test. J Endocrinol Invest.

[4] Sormani MP, De Rossi N, Schiavetti I, Carmisciano L, Cordioli C, Moiola L (2021). Disease modifying therapies and COVID-19 severity in multiple sclerosis. Ann Neurol.

[5] Louapre C, Collongues N, Stankoff B, Giannesini C, Papeix C, Bensa C (2020). Clinical characteristics and outcomes in patients with coronavirus disease 2019 and multiple sclerosis. JAMA Neurol.

[6] Peeters LM, Parciak T, Walton C, Geys L, Moreau Y, De Brouwer E (2020). COVID-19 in people with multiple sclerosis: A global data sharing initiative. Mult Scler J.

[7] Reyes S, Cunningham AL, Kalincik T, Havrdová EK, Isobe N, Pakpoor J (2021). Update on the management of multiple sclerosis during the COVID-19 pandemic and post pandemic: An international consensus statement. J Neuroimmunol.

[8] Reyes S, Ramsay M, Ladhani S, Amirthalingam G, Singh N, Cores C (2020). Protecting people with multiple sclerosis through vaccination. Pract Neurol.

[9] Ciotti JR, Valtcheva MV, Cross AH (2020). Effects of MS disease-modifying therapies on responses to vaccinations: A review. Mult Scler Relat Disord.

[10] Bar-Or A, Freedman MS, Kremenchutzky M, Menguy-Vacheron F, Bauer D, Jodl S (2013). Teriflunomide effect on immune response to influenza vaccine in patients with multiple sclerosis. Neurology.

[11] Bar-Or A, Wiendl H, Miller B, Benamor M, Truffinet P, Church M (2015). Randomized study of teriflunomide effects on immune responses to neoantigen and recall antigens. Neurol Neuroimmunol Neuroinflamm.

[12] von Hehn C, Howard J, Liu S, Meka V, Pultz J, Mehta D (2018). Immune response to vaccines is maintained in patients treated with dimethyl fumarate. Neurol Neuroimmunol Neuroinflamm.

[13] Kappos L, Mehling M, Arroyo R, Izquierdo G, Selmaj K, Curovic-Perisic V (2015). Randomized trial of vaccination in fingolimod-treated patients with multiple sclerosis. Neurology.

[14] Ufer M, Shakeri-Nejad K, Gardin A, Su Z, Paule I, Marbury TC (2017). Impact of siponimod on vaccination response in a randomized, placebo-controlled study. Neurol Neuroimmunol Neuroinflamm.

[15] Bar-Or A, Calkwood JC, Chognot C, Evershed J, Fox EJ, Herman A (2020). Effect of ocrelizumab on vaccine responses in patients with multiple sclerosis. Neurology.

[16] McCarthy CL, Tuohy O, Compston DAS, Kumararatne DS, Coles AJ, Jones JL (2013). Immune competence after alemtuzumab treatment of multiple sclerosis. Neurology.

[17] Achiron A, Mandel M, Dreyer-Alster S, Harari G, Magalashvili D, Sonis P (2021). Humoral immune response to COVID-19 mRNA vaccine in patients with multiple sclerosis treated with high-efficacy disease-modifying therapies. Ther Adv Neurol Disord.

[18] Cordonnier C, Einarsdottir S, Cesaro S, Di Blasi R, Mikulska M, Rieger C (2019). Vaccination of haemopoietic stem cell transplant recipients: Guidelines of the 2017 European Conference on Infections in Leukaemia (ECIL 7). Lancet Infect Dis.

[19] Prosperini L, Mancinelli C, Haggiag S, Cordioli C, De Giglio L, De Rossi N (2020). Minimal evidence of disease activity (MEDA) in relapsing-remitting multiple sclerosis. J Neurol Neurosurg Psychiatry.

[20] Cristiano E, Alonso R, Alvez Pinheiro A, Bacile EA, Balbuena ME, Ballario C (2018). Argentinean recommendations on the identification of treatment failure in relapsing remitting multiple sclerosis patients. J Neurol Sci.

[21] Tsantes E, Curti E, Collura F, Bazzurri V, Fiore A, Granella F (2020). Five- and seven-year prognostic value of new effectiveness measures (NEDA, MEDA and six-month delayed NEDA) in relapsing-remitting multiple sclerosis. J Neurol Sci.

[22] Roos I, Leray E, Frascoli F, Casey R, Brown JWL, Horakova D (2020). Delay from treatment start to full effect of immunotherapies for multiple sclerosis. Brain.

[23] Giovannoni G, Turner B, Gnanapavan S, Offiah C, Schmierer K, Marta M (2015). Is it time to target no evident disease activity (NEDA) in multiple sclerosis?. Mult Scler Relat Disord.

[24] De Stefano N, Giorgio A, Tintoré M, Pia Amato M, Kappos L, Palace J (2018). Radiologically isolated syndrome or subclinical multiple sclerosis: MAGNIMS consensus recommendations. Mult Scler J.

[25] Okuda DT, Siva A, Kantarci O, Inglese M, Katz I, Tutuncu M (2014). Radiologically isolated syndrome: 5-year risk for an initial clinical event. PLoS ONE.

[26] Lebrun-Frenay C, Kantarci O, Siva A, Sormani MP, Pelletier D, Okuda DT (2020). Radiologically isolated syndrome: 10-year risk estimate of a clinical event. Ann Neurol.

[27] Grzegorski T, Losy J (2020). What do we currently know about the clinically isolated síndrome suggestive of multiple sclerosis? An update. Rev Neurosci.

[28] Galetta SL (2001). The Controlled High Risk Avonex® Multiple Sclerosis trial (CHAMPS STUDY). J Neuroophthalmol.

[29] Comi G, Filippi M, Barkhof F, Durelli L, Edan G, Fernández O (2001). Effect of early interferón treatment on conversion to definite multiple sclerosis: A randomised study. Lancet.

[30] Kappos L, Polman CH, Freedman MS, Edan G, Hartung HP, Miller DH (2006). Treatment with interferon beta-1b delays conversion to clinically definite and McDonald MS in patients with clinically isolated syndromes. Neurology.

[31] Comi G, Martinelli V, Rodegher M, Moiola L, Bajenaru O, Carra A (2009). Effect of glatiramer acetate on conversion to clinically definite multiple sclerosis in patients with clinically isolated syndrome (PreCISe study): A randomised, double-blind, placebo-controlled trial. Lancet.

[32] Comi G, De Stefano N, Freedman MS, Barkhof F, Polman CH, Uitdehaag BM (2012). Comparison of two dosing frequencies of subcutaneous interferon beta-1a in patients with a first clinical demyelinating event suggestive of multiple sclerosis (REFLEX): A phase 3 randomised controlled trial. Lancet Neurol.

[33] Leist TP, Comi G, Cree BAC, Coyle PK, Freedman MS, Hartung H-P (2014). Effect of oral cladribine on time to conversion to clinically definite multiple sclerosis in patients with a first demyelinating event (ORACLE MS): A phase 3 randomised trial. Lancet Neurol.

[34] Miller AE, Wolinsky JS, Kappos L, Comi G, Freedman MS, Olsson TP (2014). Oral teriflunomide for patients with a first clinical episode suggestive of multiple sclerosis (TOPIC): A randomised, double-blind, placebo-controlled, phase 3 trial. Lancet Neurol.

[35] Polman CH, O’Connor PW, Havrdova E, Hutchinson M, Kappos L, Miller DH (2006). A randomized, placebo-controlled trial of natalizumab for relapsing multiple sclerosis. N Engl J Med.

[36] Rudick RA, Stuart WH, Calabresi PA, Confavreux C, Galetta SL, Radue E-W (2006). Natalizumab plus interferon beta-1a for relapsing multiple sclerosis. N Engl J Med.

[37] Berger JR, Fox RJ (2016). Reassessing the risk of natalizumab-associated PML. J Neurovirol.

[38] Major EO, Nath A (2016). A link between long-term natalizumab dosing in MS and PML. Neurol Neuroimmunol Neuroinflamm.

[39] Bomprezzi R, Pawate S (2014). Extended interval dosing of natalizumab: A two-center, 7-year experience. Ther Adv Neurol Disord.

[40] Petržalka M, Meluzínová E, Mojžišová H, Libertínová J, Ročková P, Němá E (2020). Effectiveness of natalizumab extended interval dosing in multiple sclerosis patients. Česká a Slov Neurol a Neurochir.

[41] Zhovtis Ryerson L, Frohman TC, Foley J, Kister I, Weinstock-Guttman B, Tornatore C (2016). Extended interval dosing of natalizumab in multiple sclerosis. J Neurol Neurosurg Psychiatry.

[42] Ryerson LZ, Foley J, Chang I, Kister I, Cutter G, Metzger RR (2019). Risk of natalizumabassociated PML in patients with MS is reduced with extended interval dosing. Neurology.

[43] Biogen A study to evaluate efficacy, safety, and tolerability of EID of natalizumab (BG00002) in participants with RRMS switching from treatment with natalizumab SID in relation to continued SID treatment- followed by extension study comprising SC and IV natalizumab administration. 2020. Report No.: NCT03689972.

[44] Freedman MS, Patry DG, Grand’Maison F, Myles ML, Paty DW, Selchen DH (2004). Treatment optimization in multiple sclerosis. Can J Neurol Sci.

[45] Kalincik T, Buzzard K, Jokubaitis V, Trojano M, Duquette P, Izquierdo G (2014). Risk of relapse phenotype recurrence in multiple sclerosis. Mult Scler J.

[46] Hirst C, Ingram G, Pearson O, Pickersgill T, Scolding N, Robertson N. (2008). Contribution of relapses to disability in multiple sclerosis. J Neurol.

[47] Kalincik T (2015). Multiple sclerosis relapses: Epidemiology, outcomes and management. A systematic review. Neuroepidemiology.

[48] Citterio A, La Mantia L, Ciucci G, Candelise L, Brusaferri F, Midgard R (2000). Corticosteroids or ACTH for acute exacerbations in multiple sclerosis. Cochrane Database Syst Rev.

[49] Richert ND, Ostuni JL, Bash CN, Leist TP, McFarland HF, Frank JA (2001). Interferon beta-1b and intravenous methylprednisolone promote lesion recovery in multiple sclerosis. Mult Scler J.

[50] Amaya-Nieto J, Girón-Luque F, Baez-Suárez Y (2017). Strongyloides stercoralis; reporte de un caso en el post-trasplante renal. Rev Med.

[51] Greaves D, Coggle S, Pollard C, Aliyu SH, Moore EM (2013). Strongyloides stercoralis infection. BMJ.

[52] Keiser PB, Nutman TB (2004). Strongyloides stercoralis in the immunocompromised population. Clin Microbiol Rev.

[53] Ghosh K, Ghosh K (2007). Strongyloides stercoralis septicaemia following steroid therapy for eosinophilia: Report of three cases. Trans R Soc Trop Med Hyg.

[54] Mejia R, Nutman TB (2012). Screening, prevention, and treatment for hyperinfection síndrome and disseminated infections caused by Strongyloides stercoralis. Curr Opin Infect Dis.

[55] Miravalle AA, Schreiner T (2014). Neurologic complications of vaccinations. En: Biller J, Ferro JM. Handbook of Clinical Neurology. Neurologic aspects of systemic disease.

[56] Schonberger LB, Bregman DJ, Sullivan-Bolyai JZ, Keelyside RA, Ziegler DW, Retailliau HF (1979). Guillain-Barré syndrome following vaccination in the National Influenza Immunization Program, United States, 1976-1977. Am J Epidemiol.

[57] Stowe J, Andrews N, Miller E (2020). Do vaccines trigger neurological diseases? Epidemiological evaluation of vaccination and neurological diseases using examples of multiple sclerosis, Guillain-Barré syndrome and narcolepsy. CNS Drugs.

[58] Karussis D, Petrou P (2014). The spectrum of post-vaccination inflammatory CNS demyelinating syndromes. Autoimmun Rev.

[59] Torisu H, Okada K (2019). Vaccination-associated acute disseminated encephalomyelitis. Vaccine.

[60] Wildemann B, Jarius S, Hartmann M, Regula JU, Hametner C (2009). Acute disseminated encephalomyelitis following vaccination against human papilloma virus. Neurology.

[61] Scheller NM, Svanström H, Pasternak B, Arnheim-Dahlström L, Sundström K, Fink K (2015). Quadrivalent HPV vaccination and risk of multiple sclerosis and other demyelinating diseases of the central nervous system. JAMA.

[62] Jakimovski D, Weinstock-Guttman B, Ramanathan M, Dwyer MG, Zivadinov R (2020). Infections, vaccines and autoimmunity: A multiple sclerosis perspective. Vaccines.

[63] Farez MF (2011). Yellow fever vaccination and increased relapse rate in travelers with multiple sclerosis. Arch Neurol.

[64] Huttner A, Eperon G, Lascano AM, Roth S, Schwob JM, Siegrist CA (2020). Risk of MS relapse after yellow fever vaccination. Neurol Neuroimmunol Neuroinflamma.

[65] Raffel J, Wakerley B, Nicholas R (2016). Multiple sclerosis. Medicina.

[66] Gross RH, Corboy JR. (2019). Monitoring, switching, and stopping multiple sclerosis diseasemodifying therapies. Contin Lifelong Learn Neurol.

[67] Reich DS, Lucchinetti CF, Calabresi PA (2018). Multiple Sclerosis. N Engl J Med.

[68] Weideman AM, Tapia-Maltos MA, Johnson K, Greenwood M, Bielekova B (2017). Meta-analysis of the age-dependent efficacy of multiple sclerosis treatments. Front Neurol.

[69] Corboy J (2021). Discontinuation of disease modifying therapies (DMTs) in multiple sclerosis (MS) (DISCOMS). (NCT03073603).

[70] Willison HJ, Jacobs BC, van Doorn PA (2016). Guillain-Barré syndrome. Lancet.

[71] Farcas P, Avnun L, Frisher S, Herishanu Y, Wirguin I (1997). Efficacy of repeated intravenous immunoglobulin in severe unresponsive Guillain-Barré syndrome. Lancet.

[72] Verboon C, Doets AY, Galassi G, Davidson A, Waheed W, Péréon Y (2019). Current treatment practice of Guillain-Barré syndrome. Neurology.

[73] Walgaard C, Jacobs BC, Lingsma HF, Steyerberg EW, Cornblath DR, van Doorn PA (2018). Second IVIg course in Guillain-Barré syndrome patients with poor prognosis (SID-GBS trial): Protocol for a double-blind randomized, placebo-controlled clinical trial. J Peripher Nerv Syst.

[74] van Doorn PA, Kuitwaard K, Walgaard C, van Koningsveld R, Ruts L, Jacobs BC (2010). IVIG treatment and prognosis in Guillain-Barré syndrome. J Clin Immunol.

[75] Hughes RAC, Swan AV, Raphael JC, Annane D, van Koningsveld R, van Doorn PA (2007). Immunotherapy for Guillain-Barre syndrome: A systematic review. Brain.

[76] Dionne A, Nicolle MW, Hahn AF (2010). Clinical and electrophysiological parameters distinguishing acute-onset chronic inflammatory demyelinating polyneuropathy from acute inflammatory demyelinating polyneuropathy. Muscle Nerve.

[77] Alessandro L, Pastor-Rueda JM, Wilken M, Querol L, Marrodán M, Acosta JN (2018). Differences between acute-onset chronic inflammatory demyelinating polyneuropathy and acute inflammatory demyelinating polyneuropathy in adult patients. J Peripher Nerv Syst.

[78] Ruts L, van Koningsveld R, van Doorn PA (2005). Distinguishing acute-onset CIDP from Guillain- Barré syndrome with treatment related fluctuations. Neurology.

[79] Chen ES, Moller DR (2011). Sarcoidosis-scientific progress and clinical challenges. Nat Rev Rheumatol.

[80] Kraaijvanger R, Janssen Bonás M, Vorselaars ADM, Veltkamp M (2020). Biomarkers in the diagnosis and prognosis of sarcoidosis: Current use and future prospects. Front Immunol.

[81] Oksanen V, Fyhrquist F, Somer H, Gronhagen-Riska C (1985). Angiotensin converting enzyme in cerebrospinal fluid: A new assay. Neurology.

[82] Dale JC, O’Brien JF (1999). Determination of angiotensin-converting enzyme levels in cerebrospinal fluid is not a useful test for the diagnosis of neurosarcoidosis. Mayo Clin Proc.

[83] Tahmoush AJ, Amir MS, Connor WW, Farry JK, Didato S, Ulhoa-Cintra A (2002). CSF-ACE activity in probable CNS neurosarcoidosis. Sarcoidosis Vasc Diffuse Lung Dis.

[84] Khoury J, Wellik KE, Demaerschalk BM, Wingerchuk DM. (2009). Cerebrospinal fluid angiotensinconverting enzyme for diagnosis of central nervous system sarcoidosis. Neurologist.

[85] Bridel C, Courvoisier DS, Vuilleumier N, Lalive PH (2015). Cerebrospinal fluid angiotensinconverting enzyme for diagnosis of neurosarcoidosis. J Neuroimmunol.

